# Stigmatisation of schizophrenia, psychosis and autism in flemish newspapers

**DOI:** 10.1192/j.eurpsy.2021.1353

**Published:** 2021-08-13

**Authors:** E. Thys, C. Struyven

**Affiliations:** Psychiatry, UPC KU Leuven, Kortenberg, Belgium

**Keywords:** Stigma, psychosis, schizophrénia, autism

## Abstract

**Introduction:**

Stigma is a major burden and impediment for treatment and recovery in serious mental disorders, especially psychotic disorders. Therefore, it has been proposed to replace the term schizophrenia by psychosis susceptibility or psychosis spectrum disorder.

**Objectives:**

We have assessed stigma in the media through a 10-year survey of Flemish daily newspapers (2008-2017) by comparing the way schizophrenia and autism are portrayed. We added the term psychosis for the years 2013-17 to assess its suitability as a less stigmatising alternative.

**Methods:**

Via the websites of the seven Flemish newspapers, we searched for all articles published between 01 Jan 2008 and 31 Dec 2017 containing the keywords autism, schizophrenia, and related terms. The collected articles (n = 5,337) were then graded to their stigmatising content. We added the term psychosis for the years 2013-17.

**Results:**

In the collected articles the coverage of autism was mostly positive, whereas the coverage of schizophrenia was predominantly negative. The contrast was very substantial (p < 0.0001) and stable over the years. The portrayal of psychosis turned out to be mostly positive in the broadsheet newspapers and mostly negative in the tabloid papers.

**Conclusions:**

The social stigma attached to schizophrenia and psychosis is poignantly reflected in the Flemish newspapers. The fact that a comparable disorder such as autism is depicted in a much more favourable way than schizophrenia indicates that a more positive image of schizophrenia is not only desirable but also achievable. Psychosis gives mixed results, a finding up for discussion.

